# Investigating the clinical validity of the guided progression analysis definition with 10–2 visual field in retinitis pigmentosa

**DOI:** 10.1371/journal.pone.0291208

**Published:** 2023-09-08

**Authors:** Shotaro Asano, Ryo Asaoka, Akio Oishi, Yuri Fujino, Hiroshi Murata, Keiko Azuma, Manabu Miyata, Ryo Obata, Tatsuya Inoue

**Affiliations:** 1 Department of Ophthalmology, Graduate School of Medicine, The University of Tokyo, Tokyo, Japan; 2 Department of Ophthalmology, Asahi General Hospital, Asahi, Chiba, Japan; 3 Department of Ophthalmology, Seirei Hamamatsu General Hospital, Shizuoka, Japan; 4 Seirei Christopher University, Shizuoka, Japan; 5 Nanovision Research Division, Research Institute of Electronics, Shizuoka University, Shizuoka, Japan; 6 The Graduate School for the Creation of New Photonics Industries, Shizuoka, Japan; 7 Department of Ophthalmology and Visual Sciences, Kyoto University Graduate School of Medicine, Kyoto, Japan; 8 Department of Ophthalmology and Visual Sciences, Nagasaki University, Nagasaki, Japan; 9 Department of Ophthalmology, Shimane University Faculty of Medicine, Izumo, Japan; 10 Department of Ophthalmology and Micro-Technology, Yokohama City University, Kanagawa, Japan; L V Prasad Eye Institute, INDIA

## Abstract

**Purpose:**

To investigate the clinical validity of the Guided Progression Analysis definition (GPAD) and cluster-based definition (CBD) with the Humphrey Field Analyzer (HFA) 10–2 test in retinitis pigmentosa (RP).

**Methods:**

Ten non-progressive RP visual fields (VFs) (HFA 10–2 test) were simulated for each of 10 VFs of 111 eyes (10 simulations × 10 VF sequencies × 111 eyes = 111,000 VFs; Dataset 1). Using these simulated VFs, the specificity of GPAD for the detection of progression was determined. Using this dataset, similar analyses were conducted for the CBD, in which the HFA 10–2 test was divided into four quadrants. Subsequently, the Hybrid Definition was designed by combining the GPAD and CBD; various conditions of the GPAD and CBD were altered to approach a specificity of 95.0%. Subsequently, actual HFA 10–2 tests of 116 RP eyes (10 VFs each) were collected (Dataset 2), and true positive rate, true negative rate, false positive rate, and the time required to detect VF progression were evaluated and compared across the GPAD, CBD, and Hybrid Definition.

**Results:**

Specificity values were 95.4% and 98.5% for GPAD and CBD, respectively. There were no significant differences in true positive rate, true negative rate, and false positive rate between the GPAD, CBD, and Hybrid Definition. The GPAD and Hybrid Definition detected progression significantly earlier than the CBD (at 4.5, 5.0, and 4.5 years, respectively).

**Conclusions:**

The GPAD and the optimized Hybrid Definition exhibited similar ability for the detection of progression, with the specificity reaching 95.4%.

## Introduction

Retinitis pigmentosa (RP) is a progressive hereditary retinal disease caused by the degeneration of rod and cone photoreceptors [1, 2]. The loss of rod and cone functions leads to constriction of the visual field (VF) and loss of fine acuity [1].

In RP, residual central VF has a negative impact on patients’ quality of life [3]. Hence, the assessment of central VF is clinically important [1, 4]. However, the clinical usefulness of the method employed to evaluate central VF progression remains to be validated in RP. The Guided Progression Analysis (GPA; Carl Zeiss Meditec, Dublin, CA, USA) software is one of the most frequently used methods for the diagnosis of VF progression [5–10]. Nonetheless, the clinical relevance of GPA definition (GPAD) [11] has not been investigated. The cluster-based definition (CBD) has been developed to improve the detection of focal VF progression in glaucoma [12]. Both the GPAD [5–10, 13–16] and CBD [5, 17] were validated in glaucomatous eyes. However, their progression detection ability have not been investigated in RP.

The primary purpose of the present study was to evaluate the usefulness of the GPAD and CBD in RP in terms of progression detection ability, and time required for the detection of VF progression. By combining these methods, we generated a new method for the detection of VF progression (Hybrid Definition) based on our previous study [17]. The clinical usefulness of the Hybrid Definition was compared with that of the GPA and CBD.

## Materials and methods

This investigation was approved by the Research Ethics Committee of the Graduate School of Medicine and Faculty of Medicine of the University of Tokyo and Kyoto University (approval number 10619). The study complied with the tenets of the Declaration of Helsinki. The patients provided written informed consent for the storage of their information in the hospital database and use for research purposes. Otherwise, based on the regulations of the Japanese Guidelines for Epidemiologic Study 2008 (issued by the Japanese Government), the study protocols did not require that each patient provide written informed consent. Instead, the protocol was posted at the outpatient clinic and/or website of the department to notify study participants. Patients or the public were not involved in the design, or conduct, or reporting, or dissemination plans of our research.

### Patients

VF (Humphrey Field Analyzer [HFA] 10–2 test) data of patients with RP were retrospectively obtained. Patients were followed up in the retina clinic at the University of Tokyo Hospital and retinal dystrophy clinic at Kyoto University Hospital between February 1, 2005 and January 31, 2020. Inclusion criteria were: (1) typical fundus findings of RP, such as bone spicule pigmentation, arteriolar attenuation, and waxy disc pallor; (2) reduction in a-wave and b-wave amplitudes or non-detectable full-field electroretinogram; (3) absence of diseases of the anterior and posterior segments of the eye other than RP that could affect VF with slit-lamp examination, intraocular pressure measurement, and funduscopy, including cataract, except for clinically insignificant cataract, and; (4) patient age ≥20 years. Patients who underwent intraocular surgery, including cataract surgery, during the observation period were excluded [18]. VF measurements were performed using HFA with the 10–2 program and the Swedish Interactive Threshold Algorithm standard. Reliability criteria for VFs were applied, including fixation losses <20% and false positive responses <15%, according to the instructions provided by the manufacturer. The initial VF was not included in the analyses to avoid the learning effect.

### Dataset 1: Test–retest variability dataset

Two HFA 10–2 tests performed within 3 months were collected, excluding those included in the Dataset 2 described below. Only one eye from each patient was randomly selected; if both eyes were eligible. In the event of multiple pairs of HFA 10–2 tests for an eye, the first pair of VFs was selected and used in the analyses. This resulted in a total of 111 pairs from 111 eyes of 111 patients (Dataset 1). Using this dataset, test–retest variations were calculated as previously described [17, 19]. In short, the total deviation (TD) values of all tested locations of the first VFs were grouped in 2-dB steps (21 groups, with TD values ranging from +3 to −38 dB). Subsequently, for each TD (first VF) group, the 95th and 99th percentiles of the TD values in the second VF test were calculated; these values were used as percentile cut-offs in the following examinations.

### Dataset 2: VF series from clinics

VF data of patients with RP who had at least 10 consecutive records of HFA 10–2 tests were collected (116 eyes). Only one eye was selected from each patient; if both eyes were eligible, the eye was randomly chosen. In case more than 10 HFA 10–2 tests were available for an eye, the first 10 VFs were selected and used in the analyses.

### Definitions of VF progression (GPAD, CBD, and Hybrid Definition)

#### GPAD

Definite VF progression is defined as a deterioration exceeding the 95th percentile (p < 0.05) of the magnitude of fluctuation at the same three or more locations in three consecutive VF tests at any test locations in VF [11, 17].

#### CBD [11, 17, 20–22]

This method was developed to overcome the possible inaccurate diagnosis of progression by the HFA 10–2 test using the point-wise linear regression. The original CBD method was developed following the anatomical mapping of the retinal nerve fibre layer to reflect the mechanism underlying glaucoma [12]. Subsequently, definite VF progression was defined as a deterioration exceeding 95 percentiles of fluctuation at the same three or more locations on three consecutive VF tests in a single sector. On the other hand, RP refers to degeneration of retinal photoreceptors which are distributed symmetrically near the fovea [23, 24]. Thus, we simply divided the HFA 10–2 VF into four quadrants, namely temporal-superior, nasal-superior, temporal-inferior, and nasal-inferior areas. Definite VF progression was then defined as a deterioration exceeding the 95th percentile of fluctuation at three or more locations in three consecutive VF tests in the same quadrant.

#### Hybrid Definition

Initially, we investigated the effects of various parameters in the GPAD and CBD on the specificity (as detailed in the next section): number of test locations (from two to five); number of consecutive VF tests (from two to three), and the percentile cut-off (either 95% or 99%). Based on Fisher’s proposal for the standard level of significance, we hypothesized that the ideal specificity of a test is 95% [25]. Subsequently, we combined the GPAD and CBD using varied parameters, in an attempt to identify a method with specificity closest to 95%. Tested combinations in the current study were listed in **Table 1**. The identified combination was termed Hybrid Definition.

**Table 1:** Combinations of parameters.

Pattern	Number of consecutive VF tests	Number of test locations	Cut-off values
1	2	≥2	95%
2	2	≥3	95%
3	2	≥4	95%
4	2	≥5	95%
5	3	≥2	95%
6	3	≥3	95%
7	3	≥4	95%
8	3	≥5	95%
9	2	≥2	99%
10	2	≥3	99%
11	2	≥4	99%
12	2	≥5	99%
13	3	≥2	99%
14	3	≥3	99%
15	3	≥4	99%
16	3	≥5	99%

VF, visual field.

### Assessment of the specificity of GPAD, CBD, and Hybrid Definition using simulated non-progressive VF series derived from Dataset 1

Initially, non-progressive series of HFA 10–2 tests were simulated, as we previously described [17, 26, 27]. Briefly, a covariance matrix was created using a test–retest dataset, which was composed of the first and second HFA10-2 tests performed within 3 months in the Dataset 1. As a result, 10 sets of 10 VFs were simulated for each eye (a total of 10 sets × 10 VF series × 116 eyes = 11,600 VFs). This simulated stable non-progressive VF dataset was used to calculate the specificities of the GPAD, CBD, and Hybrid Definition for the detection of VF progression. Specificity is defined as the ratio of true negatives to the total number of negative cases in the data: Specificity = (True negative) / (True negative + False positive). The term (True negative + False positive) is also known as the condition negative, which refers to the actual negative cases in the data. In the current study, the simulated VF series assumed non-progressive VFs with randomly generated variability based on reproducibility, so all the records belonged to the condition negative group. True negatives are the cases that are correctly classified as negative. As explained above, all the VF records generated by simulation were classified as the condition negative group, because the variability of the VFs was randomly generated according to the fluctuation of reproducibility. Therefore, the number of simulated VF series was equal to the condition negative group (True negative and False positive). The number of cases classified as negative (non-progressive) by the proposed tests in the current study were counted as true negatives, since all the cases in the simulated data were negative by definition. Thus, the probability of being diagnosed as ‘non-progressive’ in this ‘non-progressive’ simulated VF series is equal to specificity.

### Evaluation of the accuracy of GPAD, CBD and Hybrid Definition using Dataset 2

Using Dataset 2, the performance of the GPAD, CBD and Hybrid Definition was evaluated through three surrogate measures: (1) true positive rate (the proportion of both progressing [PBP]); (2) true negative rate (proportion of both not progressing [PBNP]); and (3) false positive rate (proportion of inconsistent progression [PIP]), as we previously reported [17, 28–30]. Details of these surrogate measures are provided below.

(1) PBP (i.e., a surrogate measure for true positive rate): the probability that both the complete series of VFs (VF_1_-_10_) and each of the shorter series of VFs (from VF_1-4_ to VF_1_-_9_) were classified as progressive.

(2) PBNP (i.e., a surrogate measure for true negative rate): the probability that the complete series of VFs (VF_1_-_10_) and each of the shorter series of VFs (from VF_1-4_ to VF_1-9_) were classified as not progressive.

(3) PIP (i.e., a surrogate measure for false positive rate): the probability that each of the shorter series of VFs (from VF_1_-_4_ to VF_1_-_9_) was classified as progressive when the complete series of VFs (VF_1-10_) was classified as not progressive.

### Evaluation of the time required for the detection of progression with GPAD, CBD, and Hybrid Definition using Dataset 2

Furthermore, we conducted Kaplan–Meier survival analysis, as we previously described [17, 28–30]. The time required for the detection of progression was compared using the log-rank test.

All statistical analyses were carried out using the statistical program *R* software (version 3.6.3; http://www.r-project.org/). P-values obtained in multiple comparisons were corrected using the Hochberg correction.

## Results

Demographic data of Dataset 1 (test–retest variability dataset) are shown in Table 2. Demographic data of Dataset 2 (VF series from clinics) are shown in Table 3. Baseline mean deviation, follow-up period between VF_1_ and VF_10_, and the mean deviation progression rate, were −16.0 ± 8.9 dB (mean ± standard deviation), 8.9 ± 2.1 years, and −0.54 ± 0.27 dB/year, respectively. The interval of VF examination in Dataset 2 was 10.8 ± 7 months. In the Dataset 2, 85 eyes out of 116 eyes (73.3%) were diagnosed as progressive with GPAD. Likewise, 73 eyes out of 116 eyes (62.9%) and 87 eyes out of 116 eyes (75.0%) were diagnosed as progressive with CBD and the Hybrid definition, respectively.

**Table 2:** Demographic data of Dataset 1 (test–retest variability dataset for simulated VF series).

Number of eyes	111
Number of patients	111
Age (years), mean ± SD	50.0 ± 13.9
MD (first VF, dB), mean ± SD	−16.1 ± 7.2
MD (second VF, dB), mean ± SD	−15.8 ± 7.2

MD, mean deviation; SD, standard deviation; VF, visual field.

**Table 3:** Demographic data of Dataset 2 (real VF series from clinics).

Number of eyes	116
Number of patients	116
Age (years), mean ± SD	46.3 ± 14.0
MD (first VF, dB), mean ± SD	−16.0 ± 8.9
Follow-up (years), mean ± SD	8.9 ± 2.1
MD progression rate (dB/y), mean ± SD	−0.54 ± 0.27

MD, mean deviation; SD, standard deviation; VF, visual field.

The specificities of the GPAD and CBD are shown in Table 4. The specificity of GPAD and CBD was 95.4% and 98.5%, respectively. Specificity values obtained by altering the number of test locations, number of VF sequences, and percentile cut-off values (95% and 99%, respectively) of the GPAD and CBD are shown in Table 4.

**Table 4:** Specificities of GPAD and CBD.

95% cut-off	GPAD (any location approach)				CBD (within a cluster approach)			
	≥2 locations	≥3	≥4	≥5	≥2 locations	≥3	≥4	≥5
2 consecutive VF tests	74.1	85.5	91.4	94.8	84.2	94.4	97.9	99.3
3 consecutive VF tests	89.9	95.4	97.7	98.9	94.2	98.5	99.5	99.9
99% cut-off	Any location				Cluster-based			
	≥2 locations	≥3	≥4	≥5	≥2 locations	≥3	≥4	≥5
2 consecutive VF tests	92.0	96.6	98.4	99.3	95.6	99.0	99.7	99.9
3 consecutive VF tests	97.5	99.2	99.7	99.9	98.8	99.8	100.0	100.0

CBD, cluster-based definition; GPAD, Guided Progression Analysis definition; VF, visual field.

Table 5 shows the specificity values following the combination of the GPAD (any location approach) and CBD (within a cluster approach). The patterns correspond to those shown in Table 1. Specificity of 95.0% was observed when the following two criteria were combined: (1) GPAD with four or more locations in three consecutive VF tests with a 95% cut-off (pattern 10); and (2) CBD approach with three or more locations in two consecutive VF tests in the same quadrant with a 99% cut-off (pattern 9). This combination was selected as the Hybrid Definition.

**Table 5:** Specificity values following the combination of the GPAD and CBD.

		CBD (within a cluster approach)															
	Patterns	1	2	3	4	5	6	7	8	9	10	11	12	13	14	15	16
GPAD (any location approach)	1	74.1	74.1	74.1	74.1	74.1	74.1	74.1	74.1	74.1	74.1	74.1	74.1	74.1	74.1	74.1	74.1
	2	82.3	85.5	85.5	85.5	84.8	85.5	85.5	85.5	85.2	85.5	85.5	85.5	85.5	85.5	85.5	85.5
	3	84.0	90.9	91.4	91.4	89.5	91.4	91.4	91.4	90.5	91.4	91.4	91.4	91.3	91.4	91.4	91.4
	4	84.2	93.2	94.6	94.8	91.8	94.5	94.7	94.8	93.1	94.7	94.8	94.8	94.4	94.7	94.8	94.8
	5	81.9	88.0	89.4	89.8	89.9	89.9	89.9	89.9	88.4	89.7	89.9	89.9	89.9	89.9	89.9	89.9
	6	84.0	92.5	94.6	95.2	93.5	95.4	95.4	95.4	93.2	95.1	95.4	95.4	95.3	95.4	95.4	95.4
	7	84.2	93.9	96.6	97.5	94.1	97.5	97.7	97.7	94.7	97.3	97.7	97.7	97.3	97.7	97.7	97.7
	8	84.2	94.3	97.5	98.5	94.2	98.3	98.9	98.9	95.3	98.3	98.8	98.9	98.2	98.9	98.9	98.9
	9	82.9	90.1	91.6	91.9	89.3	92.6	91.9	92.0	92.0	92.0	92.0	92.0	92.0	92.0	92.0	92.0
	10	84.2	93.4	95.9	96.5	92.6	95.9	96.5	96.6	**95.0**	96.6	96.6	96.6	96.3	96.6	96.6	96.6
	11	84.2	94.2	97.3	98.2	93.7	97.5	98.3	98.4	95.6	98.3	98.4	98.4	97.8	98.4	98.4	98.4
	12	84.2	94.3	97.7	98.9	94.0	98.1	99.0	99.2	95.6	98.8	99.2	99.3	98.4	99.2	99.3	99.3
	13	84.0	93.4	96.2	97.2	93.6	96.8	97.4	97.5	94.9	97.1	97.4	97.4	97.5	97.5	97.5	97.5
	14	84.2	94.2	97.5	98.7	94.2	98.2	99.0	99.2	95.6	98.5	99.1	99.1	98.7	99.2	99.2	99.2
	15	84.2	94.4	97.8	99.1	94.2	98.5	99.4	99.7	95.6	98.9	99.6	99.7	98.8	99.6	99.7	99.7
	16	84.2	94.4	97.8	99.3	94.2	98.5	99.5	99.9	95.6	99.0	99.7	99.9	98.8	99.8	99.9	99.9

The underlined number in bold represents the combination that reached a specificity of 95.0%.

**Fig 1:**
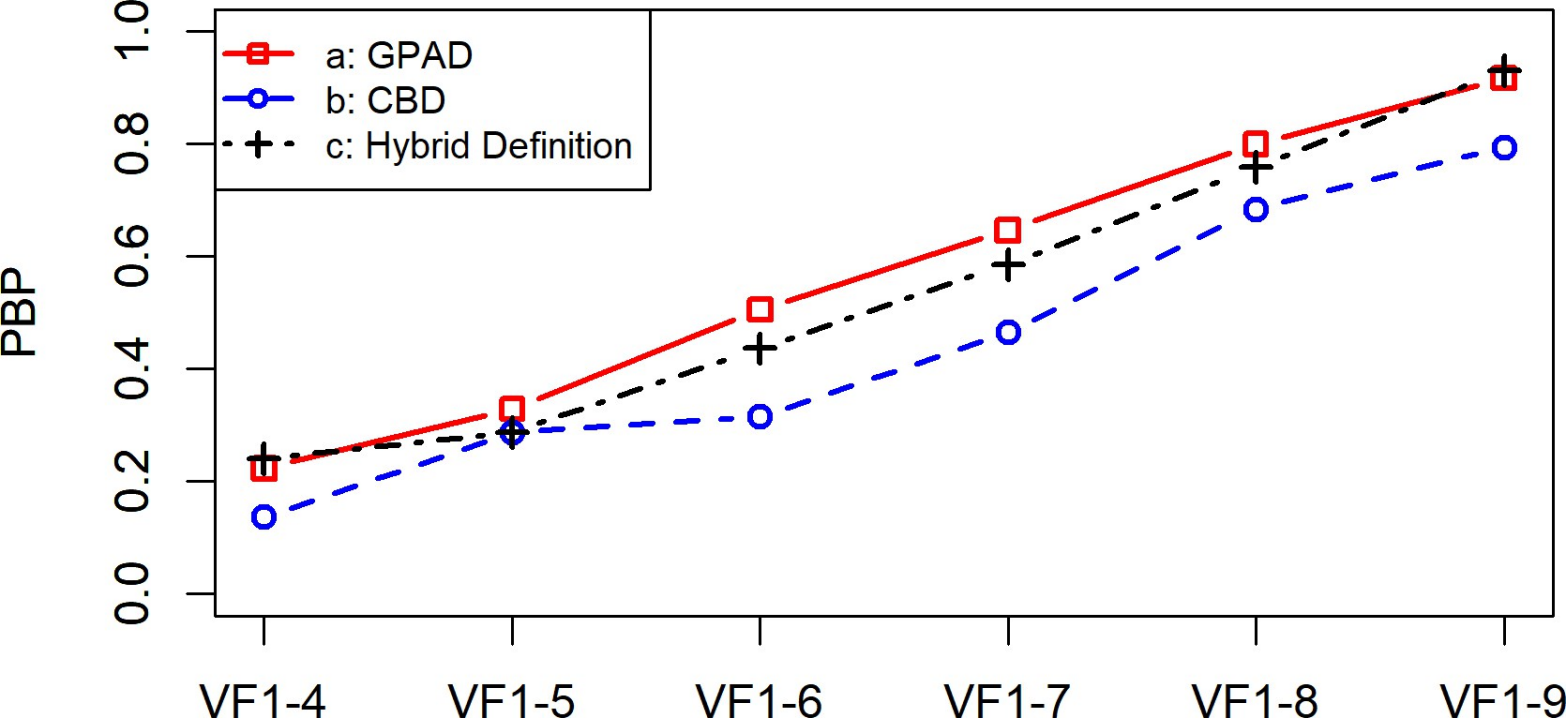
Rates of the proportion of both progressing (PBP) with the GPAD, CBD, and Hybrid Definition. There were no statistically significant differences in PBP values between the three methods. CBD, cluster-based definition; GPAD, Guided Progression Analysis definition; VF, visual field.

**Fig 2:**
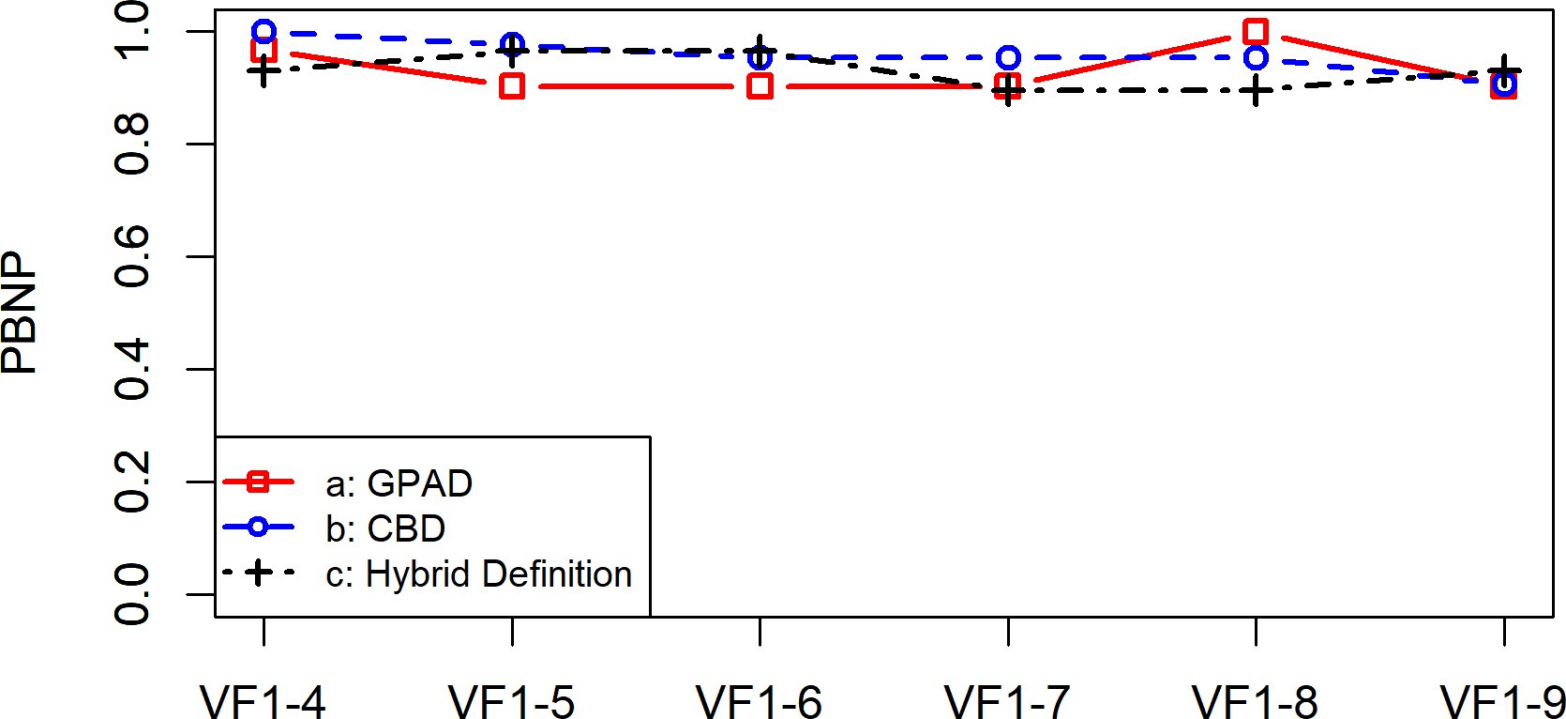
Rates of the proportion of both not progressing (PBNP) with the GPAD, CBD, and Hybrid Definition. There were no statistically significant differences in PBNP values between the three methods. CBD, cluster-based definition; GPAD, Guided Progression Analysis definition; VF, visual field.

**Fig 3:**
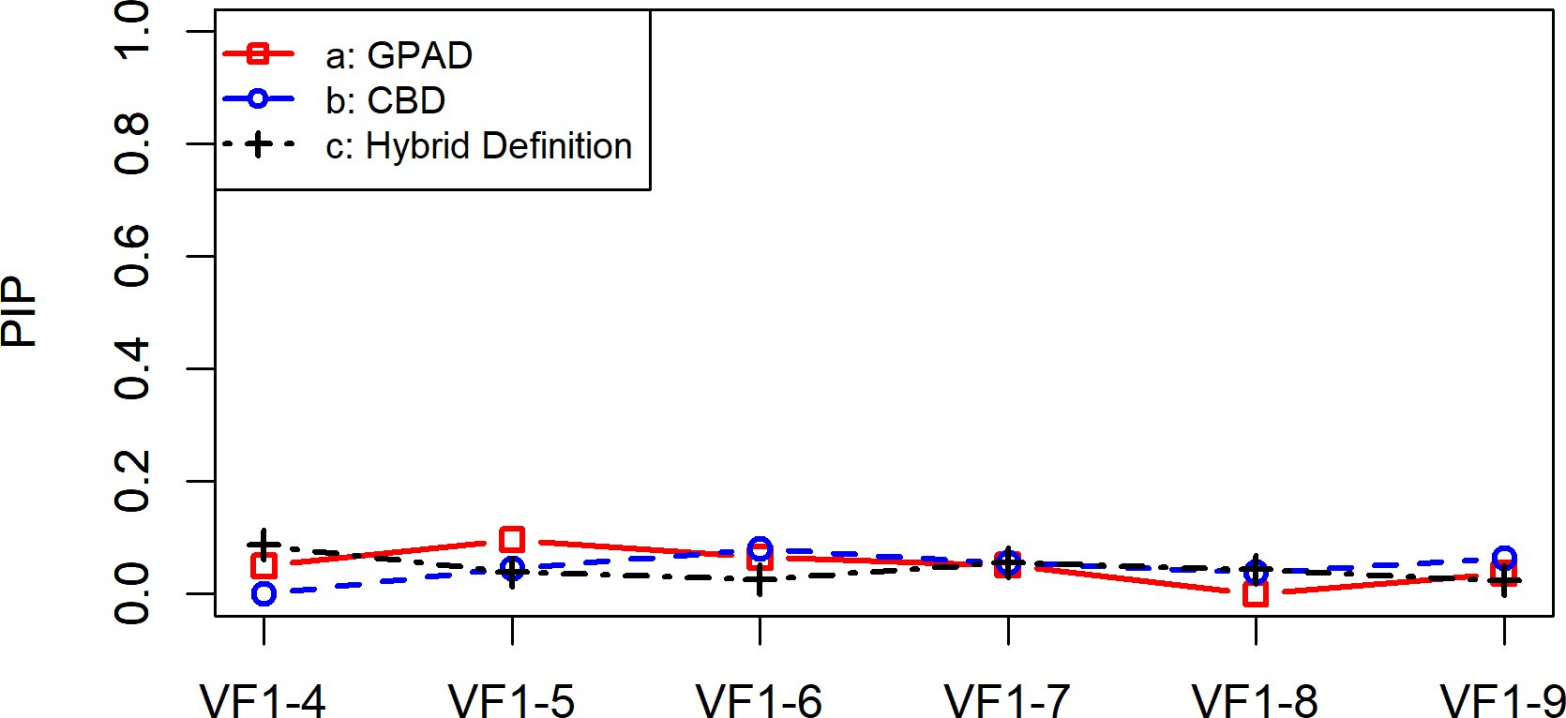
Rates of the proportion of inconsistent progressing (PIP) with the GPAD, CBD, and Hybrid Definition. There were no statistically significant differences in PIP values between the three methods. CBD, cluster-based definition; GPAD, Guided Progression Analysis definition; VF, visual field.

**Fig 1** shows the PBP values for the GPAD, CBD, and Hybrid Definition; they varied between 0.22 (VF_1-4_) and 0.92 (VF_1-9_) with GPAD, 0.14 (VF_1-4_) and 0.79 (VF_1-9_) with CBD, and 0.24 (VF_1-4_) and 0.93 (VF_1-9_) with the Hybrid Definition, respectively. There were no significant differences among the PBP values of the three methods (all p values > 0.05, in a Wilcoxon signed rank test adjusted for multiple comparisons using the Hochberg correction). **Fig 2** shows the PBNP values for the GPAD, CBD, and Hybrid Definition; they varied between 0.90 (VF_1-5_, VF_1-6_, VF_1-7_, and VF_1-9_) and 1.00 (VF_1-8_) with GPAD, 0.91 (VF_1-9_) and 1.00 (VF_1-4_) with CBD, and 0.90 (VF_1-7_ and VF_1-8_) and 0.97 (VF_1-5_ and VF_1-6_) with the Hybrid Definition, respectively. There were no significant differences among the PBNP values of the three methods (all p values > 0.05, in a Wilcoxon signed rank test adjusted for multiple comparisons using the Hochberg correction). **Fig 3** shows the PIP values for the GPAD, CBD, and Hybrid Definition; they varied between 0 (VF_1-8_) and 0.10 (VF_1-5_) with GPAD, 0 (VF_1-4_) and 0.08 (VF_1-6_) with CBD, and 0.02 (VF_1-9_) and 0.09 (VF_1-4_) with the Hybrid Definition, respectively. There were no significant differences among the PIP values of the three methods (all p values > 0.05, in a Wilcoxon signed rank test adjusted for multiple comparisons using the Hochberg correction).

**Fig 4:**
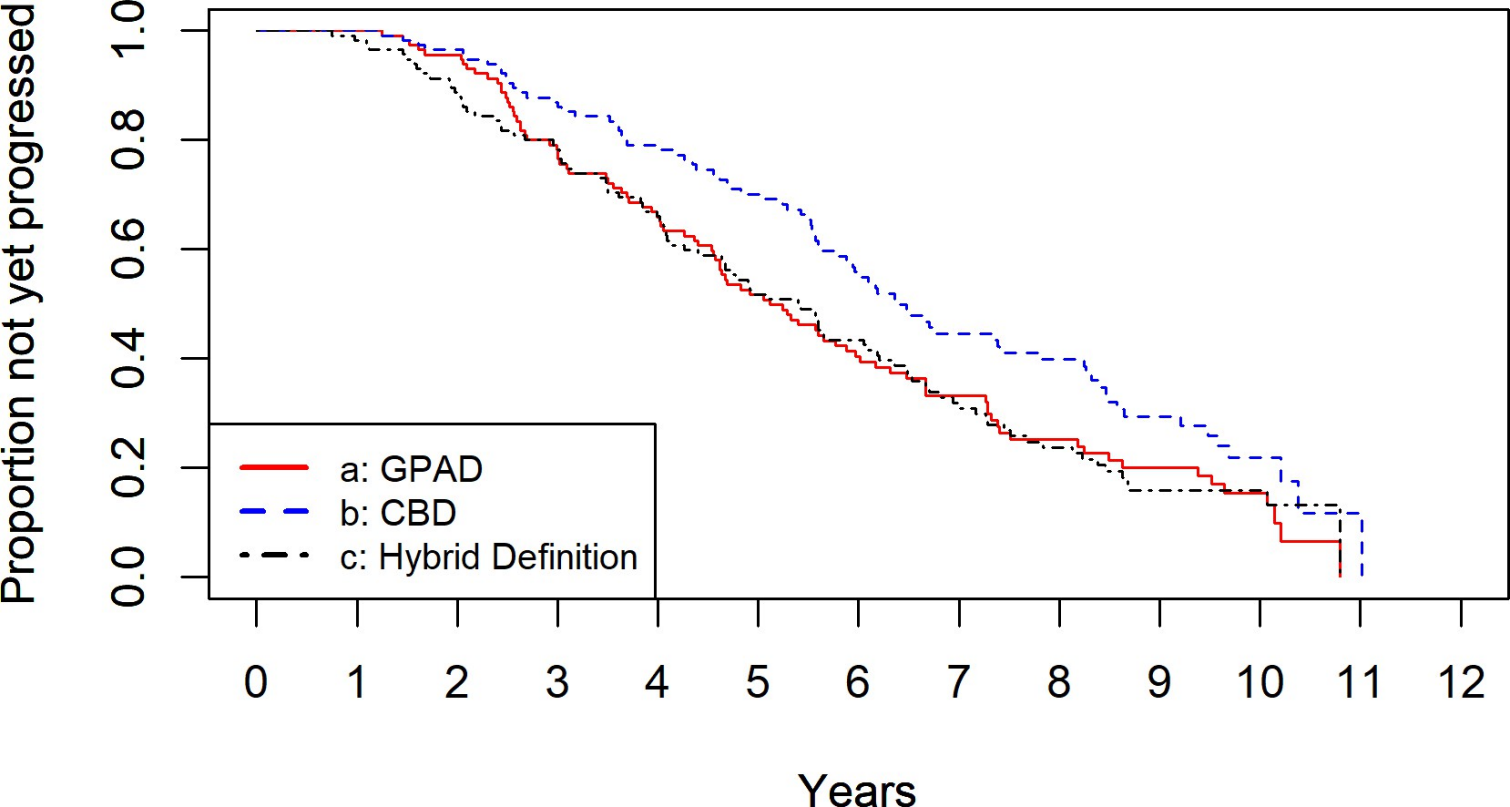
Kaplan–Meier survival analysis with the GPAD, CBD, and Hybrid Definition. GPAD and Hybrid Definition detected progression significantly earlier than the CBD (both p-values = 0.020). There was no statistically significant difference between the GPAD and Hybrid Definition (p = 1.0). CBD, cluster-based definition; GPAD, Guided Progression Analysis definition.

**Fig 4** shows the results of the Kaplan–Meier survival analysis. The mean (± standard deviation) time required to reach the diagnosis of progression with each method was 4.5 ± 2.0, 5.0 ± 2.2, and 4.5 ± 2.3 years for the GPAD, CBD, and Hybrid Definition, respectively. The survival rates of GPAD at 9, 10, and 11 years of follow-up were 0.20, 0.16, and 0. Likewise, those of CBD were 0.29, 0.22, and 0.12, and those of the Hybrid definition were 0.16, 0.16 and 0, respectively. The log-rank test results indicated that the GPAD and Hybrid Definition detected progression significantly earlier than the CBD (both p-values = 0.020, based on log-rank tests adjusted for multiple comparisons using the Hochberg correction). There was no statistically significant difference between the GPAD and Hybrid Definition (p = 1.0, based on log-rank tests adjusted for multiple comparisons using the Hochberg correction).

## Discussion

In the present study, the clinical usefulness of the GPAD and CBD for the detection of progression was investigated in eyes with RP. Initially, the specificity of each method was calculated using simulated non-progressive VF sequences (derived from a test–retest dataset [**Dataset 1 in S1 File**]) the values were 95.4% and 98.5%, respectively. Next, we investigated the effects of varying parameters (i.e., number of test locations, VF sequences, and percentile cut-off value) of the GPAD and CBD, and combined the two definitions in an attempt to identify a method (Hybrid Definition) with specificity approaching 95.0%. Subsequently, using longitudinal real VF series (**Dataset 2 in S2 File**), the usefulness of these definitions was investigated through surrogate measures of the true positive rate (PBP), true negative rate (PBNP), false positive rate (PIP), and Kaplan–Meier survival analysis. The GPAD, CBD, and Hybrid Definition did not show significant differences in PBP, PIP, and PBNP. The GPAD and Hybrid Definition detected progression significantly earlier than the CBD.

This is the first study to validate the specificity and progression detection ability of the GPAD in RP. The present study revealed that the specificity of the GPAD and CBD was 95.4% and 98.5%, respectively. Considering that the ideal specificity is 95.0% [25], there is a possibility that these methods are not optimal (i.e., excessively conservative) for the detection of VF progression in RP. The Hybrid Definition exhibited a specificity value of 95.0% (**Table 4**). Previously, in eyes with glaucoma, we experienced an improved detectability of VF progression with the Hybrid Definition approach versus the GPAD and CBD; PBP was significantly higher with the Hybrid Definition versus the GPAD and CBD [17]. In contrast, in the present study, the Hybrid Definition failed to show improved usefulness compared with the GPAD. The GPAD, CBD, and Hybrid Definition did not show significant differences in PBP (**Fig 1**), PIP (**Fig 2**), and PBNP (**Fig 3**).

In our previous study conducted on glaucomatous eyes, the time required for the detection of VF progression was significantly shorter (by approximately 1 year) with the Hybrid Definition (3.1 ± 2.0 years) than with the GPAD (4.1 ± 2.2 years) and CBD (4.2 ± 2.2 years). However, in this study, the Hybrid Definition detected VF progression significantly earlier (4.5 ± 2.3 years) than the CBD (5.0 ± 2.2 years), but not earlier than the GPAD (4.5 ± 2.0 years) (**Fig 4**). This may be attributed to the specificity of GPAD in the present study (95.4%); this was closer to 95% compared with that recorded in our previous study (99.6%) [17]. Hence, the difference between the GPAD and Hybrid Definition was minimized. These results validate the usefulness of the GPAD for the assessment of VF progression in RP at the clinical setting, and indicate that CBD may not be an appropriate approach to detect early VF progression. Of note, most previous studies in RP used trend analyses, such as the mean deviation, to evaluate VF progression [31, 32]. The current results suggest it is also adequate to use GPAD in RP. The comparison between performance of trend analysis (e.g. mean deviation) and event analysis (e.g. GPAD and CBD) in eyes with RP is expected in the future study.

In the present study, we divided the HFA 10–2 test into four quadrants based on a previous study which suggested that retinal photoreceptor cells (rods and cones) were symmetrically distributed near the fovea in primates [23]. Similarly, Zhang et al. did not report differences in cone density in the superior/nasal, superior/temporal, inferior/nasal, and inferior/temporal quadrants measured with an adaptive optics scanning laser ophthalmoscope [24]. Nonetheless, Elsner et al. showed that the temporal and nasal meridians have higher cone densities than the inferior and superior meridians in healthy young adults [33]. Therefore, there may be some differences in the manner of progression in each quadrant, which may have a non-negligible effect on the clustering result in the CBD. This hypothesis warrants further investigation in a future study.

There are some limitations in the present study. Firstly, we did not consider the genetic information of each patient. Recently, studies have highlighted the benefit of personalized medicine (e.g., gene therapy) in RP [34, 35]. Thus, information on the VF progression pattern relating to certain genetic forms of RP may be beneficial in the management of RP. Secondly, Dataset 2 was collected retrospectively. In our previous study conducted on glaucomatous eyes, the Hybrid Definition was defined as the combination of following two criteria; (1) an any-location approach of three or more locations in two consecutive VF tests with a 95% cut-off, and; (2) a cluster-based approach of two or more locations in two consecutive VF tests in the same sector, with a 95% cut-off. A prospective reproducible examination with larger sample size is desirable to validate the present findings to figure out the ideal combination of criteria to generate the Hybrid definition.

In conclusion, the clinical usefulness of the GPAD and CBD was investigated using a simulated non-progressive VF series and longitudinal VF sequences from clinics. The specificity of the GPA and CBD was 95.4% and 98.5%, respectively. The Hybrid Definition achieved a specificity value of 95.0%. The ability of the Hybrid Definition for the detection of progression was not significantly different from that of the GPAD, though it was significantly better than that of the CBD. These results were in favor of clinical validity of the GPAD for the detection of progression in RP.

## Supporting information

S1 File Dataset 1 analysed in the current study. (CSV)

S2 File Dataset 2 analysed in the current study. (CSV)
